# Exact Solutions Modelling Nonlinear Atmospheric Gravity Waves

**DOI:** 10.1007/s00021-023-00842-3

**Published:** 2023-12-20

**Authors:** David Henry

**Affiliations:** 1 Faculty of Mathematics, University of Vienna, Oskar-Morgenstern-Platz 1, 1090 Vienna, Austria; 2 Present Address: School of Mathematical Sciences, University College Cork, Western Road, Cork, Ireland

**Keywords:** Atmospheric waves, Mountain waves, Exact solution, 86A10, 35Q86, 76U60

## Abstract

Exact solutions to the governing equations for atmospheric motion are derived which model nonlinear gravity wave propagation superimposed on atmospheric currents. Solutions are explicitly prescribed in terms of a Lagrangian formulation, which enables a detailed exposition of intricate flow characteristics. It is shown that our solutions are well-suited to modelling two distinct forms of mountain waves, namely: trapped lee waves in the Equatorial *f*-plane, and vertically propagating mountain waves at general latitudes.

## Introduction

This paper presents new exact solutions which model nonlinear atmospheric gravity waves. The most general form of this exact solution represents three-dimensional motion corresponding to a wave-like term superimposed on a mean vertical and horizontal wind drift (in a fixed vertical plane), with a variable transverse wind flowing orthogonal to this plane. The solutions are prescribed explicitly in a Lagrangian framework. In general, exact solutions offer an invaluable insight into the mathematical structure of any given problem. From a physical perspective, while exact solutions are idealised representations of fluid flows, they provide a foundation upon which more realistic and observable flows may be constructed. In the past decade, there has been a flurry of mathematical activity generating exact solutions for homogeneous inviscid fluid flows in the context of geophysical water waves (see [4, 10, 17, 19], and the reference therein) building on Gerstner’s solution. In the context of the solution presented in this paper, we note that the inclusion of a transverse current for ocean flows was first achieved in [15, 16].

There is a dearth of similar exact solutions in the more intricate setting of compressible atmospheric flows. This is not surprising since the jump in complexity from modelling water waves, to modelling waves in the atmosphere, is vast. In addition to satisfying the Euler equation and the equation of mass conservation, solutions are also required to satisfy the equation of state for the air, and the first law of thermodynamics see [7–9]. Moreover, very few papers are devoted to nonlinear atmospheric flows in Lagrangian coordinates, although where Lagrangian solutions do exist the level of insight into the resulting kinematical and flow properties which is readily available (see, for example, [5]) is far richer than for solutions which are derived within the Eulerian setting (cf. [20, 22, 23]). An exact nonlinear Lagrangian solution for incompressible inviscid air flow on an interface between two regions of constant density was derived in [24], while recently in [6] this work was significantly generalised to accommodate a continuous density variation as well as a tilted direction of wave propagation.

In this paper we generalise the two-dimensional flow in [6] to three dimensions by introducing variable transverse currents into the exact solution, and further generalise the structural complexity of the governing equations by incorporating the Coriolis effects of the Earth’s rotation. We consider waves whose physical scale is such that we may neglect the Earth’s sphericity, but we aim to retain the effects of the Earth’s rotation by incorporating Coriolis forces in an *f*-plane approximation. The conclusions of this paper are:

(i) There exist exact atmospheric gravity wave solutions to the *f*-plane approximation in the equatorial region. These solutions consist of nonlinear waves propagating along the equator superimposed on a constant mean zonal current, and a variable transverse current flowing meridionally. However, it is not possible to incorporate a mean vertical current into this type of explicit solution.
(ii) Neglecting Earth’s rotation, one can incorporate mean vertical currents into an exact solution. Atmospheric gravity waves may propagate both vertically and horizontally, due to air compressibility and stratification. In atmospheric flows the horizontal velocity component is usually many orders of magnitude larger than the vertical [31], which is often neglected. However, situations exist where this is not the case, such as mountain waves.

The first scenario described in (i), and presented in Sect. 4.1, offers a model for trapped lee waves, whereby a moderate wind is forced upwards by a mountain, in the process overshooting the mountain peak. This upward wave propagation may then be arrested, and deflected downwards, by a variety of factors, such as the existence of a thermal inversion layer (which acts like a lid on the updraft), or by the presence of a unstable layer aloft comprising much stronger winds, see [26, 32]. The affected air parcels alternately fall and rise, due to cooling and heating effects (and the associated interplay between competing gravity and buoyancy forces), thereby initiating a series of oscillations.

Since the wind energy is trapped within the stable layer, these mountain waves propagate for long distances (from tens, to hundreds, of kilometres downstream [28]) downwind on the lee side of the mountain, and at altitudes close to the mountain peak.

The second scenario modelled in (ii), and described in Sect. 4.2, generalises a recently derived solution [6] which models upward-propagating mountain waves by incorporating a variable transverse current. Upward propagating mountain waves feature a vertical velocity component which is significant, and they can occur where there is no thermal inversion layer above the mountain peak, and when the wind speed also does not increase significantly with altitude. In this setting, and when the air temperature and density also decrease above the mountain peak, atmospheric waves propagate upwards into the lower stratosphere [29] (unless they steepen and break before reaching the tropopause) with wave amplitudes increasing with ascending altitude.

Mountain waves typically comprise a laminar layer of smooth waves, with layers of clear air turbulence aloft and overturning eddies (rotors) beneath. The exact solutions we present model flow in the smooth laminar layer, satisfying the nonlinear governing equations for inviscid compressible flow that we now present.

## Preliminaries

The physical scales relating to the waves being considered enable the sphericity of the earth to be neglected [31], therefore the governing equations will presented in terms of a Cartesian coordinate frame with the $x'$-axis in the horizontal direction of wave propagation, the $y'$-axis lying horizontally orthogonal to it, and the $z'$-axis pointing vertically upwards. Then $(u',v',w')$ is the velocity field of the fluid, $\rho '$ is the density of the fluid, $P'$ is the atmospheric pressure, ${\mathcal {T}}'$ is the (absolute) temperature, and $g'=9.8$ms^-2^ is the gravitational acceleration at the surface of the earth. Primes denote physical variables throughout, and these will subsequently be removed when variables are non-dimensionalised.

The full generality of the model being considered incorporates the effects of Earth’s rotation, with Earth regarded as a perfect sphere of radius $R=6378$ km with constant rotational speed of $\Omega '=7.3\times 10^{-5}$ rad/s. Then the governing equations (see [18]) for equatorial flows in the *f*-plane approximation take the form of the Euler equations

2.1a $$\begin{aligned} \frac{\partial u'}{\partial t'}+ u' \frac{\partial u'}{\partial x'} +v\frac{\partial u'}{\partial y'} +w'\frac{\partial u'}{\partial z'}+2 \Omega ' w'&=-\frac{1}{\rho '} \frac{\partial P'}{\partial x'}, \end{aligned}$$

2.1b $$\begin{aligned} \frac{\partial v'}{\partial t'}+ u' \frac{\partial v'}{\partial x'} +v\frac{\partial v'}{\partial y'} +w'\frac{\partial v'}{\partial z'}&=-\frac{1}{\rho '} \frac{\partial P'}{\partial y'} \end{aligned}$$

2.1c $$\begin{aligned} \frac{\partial w'}{\partial t'}+ u' \frac{\partial w'}{\partial x'} +v\frac{\partial w'}{\partial y'} +w'\frac{\partial w'}{\partial z'}-2 \Omega ' u'&=-\frac{1}{\rho '} \frac{\partial P'}{\partial z'}-g'. \end{aligned}$$

together with the equation for mass conservation

2.2 $$\begin{aligned} \frac{\partial \rho '}{\partial t'}+u'\frac{\partial \rho '}{\partial x'}+v'\frac{\partial \rho '}{\partial y'}+w'\frac{\partial \rho '}{\partial z'} +\rho '\left( \frac{\partial u'}{\partial x'}+\frac{\partial v'}{\partial y'}+\frac{\partial w'}{\partial z'}\right) =0, \end{aligned}$$

the equation of state for an ideal gas

2.3 $$\begin{aligned} P'=\rho ' {\mathfrak {R}}' {\mathcal {T}}', \end{aligned}$$

and the first law of thermodynamics

2.4 $$\begin{aligned} c_p'\left( \frac{\partial {\mathcal {T}}'}{\partial t'}+ u' \frac{\partial {\mathcal {T}}'}{\partial x'} +v\frac{\partial {\mathcal {T}}'}{\partial y'} +w'\frac{\partial {\mathcal {T}}'}{\partial z'} \right) -\frac{1}{\rho '}\left( \frac{\partial P'}{\partial t'}+ u' \frac{\partial P'}{\partial x'} +v\frac{\partial P'}{\partial y'} +w'\frac{\partial P'}{\partial z'} \right) =Q'. \end{aligned}$$

Here $Q'$ is the heat-source term, $c_p'\approx 1000$m^2^s^-2^K^-1^ is the specific heat of dry air at an atmospheric pressure of 1000mb, and ${\mathfrak {R}}'\approx 297$m^2^s^-2^K^-1^ is the gas constant for dry air.

The applicability of the inviscid governing equations (2.1)–(2.4) to modelling atmospheric waves, and in particular mountain waves, is discussed in greater detail in [6, 28, 29, 32, 34]. The Euler equations given in (2.1) correspond to an *f*-plane approximation in the equatorial region whereby the $x'$ and $y'$ axes point in the longitudinal and latitudinal directions, respectively. This model is valid when latitudinal variation about the equator is relatively small, and can be taken as fixed. The terms involving $\Omega$ in (2.1) capture the Coriolis effects of the Earth’s rotation, and these effects can be neglected by setting $\Omega =0$ (which is applicable for waves whose physical scales are such that the effects of the Earth’s rotation are insignificant). The resulting Euler equations (2.1) are then applicable to general locations on Earth, and are not restricted to the equatorial region.

The equations of motion can be non-dimensionalised by introducing new variables $t,x,y,z,u,v,w,\rho ,P,{\mathcal {T}}$ by way of the transformation

2.5 $$\begin{aligned} t'&=\left( L'/U'\right) t, \ (x',y',z')=L'(x,y,z), \ (u',v',w')=U'(u,v,w), \nonumber \\ \rho '&={\bar{\rho }}'\rho , \ P'={\bar{\rho }}'U'^2P, \ {\mathcal {T}}'=\left( U'^2/{\mathcal {R}}'\right) {\mathcal {T}}. \end{aligned}$$

Here ${\bar{\rho }}'\approx 1$ kg m^-3^ is the average density in the lower troposphere [33], and $L'$ and $U'$ are characteristic physical scales for the wave length and speed. In the setting of short mountain waves, suitable choices are $L'=2$ km and $U'=20$ m s^-1^, leading to $L'/U'\approx 100$ s, ${\bar{\rho }}'U'^2\approx 10^{-2}$ atm, and $(U'^2/{\mathfrak {R}}')\approx 1^{\circ }K$. We note that 2 km is smaller than current grid spacings in global weather and climate prediction models [30], further motivating the pursuit of a theoretical approach in modelling and understanding these atmospheric gravity waves.

The change of variables (2.5) leads to the nondimensionalised governing equations

2.6a $$\begin{aligned}&\frac{\partial u}{\partial t}+ u \frac{\partial u}{\partial x} +v\frac{\partial u}{\partial y} +w\frac{\partial u}{\partial z}+2 \Omega w =-\frac{1}{\rho } \frac{\partial P}{\partial x}, \end{aligned}$$

2.6b $$\begin{aligned}&\frac{\partial v}{\partial t}+ u \frac{\partial v}{\partial x} +v\frac{\partial v}{\partial y} +w\frac{\partial v}{\partial z} =-\frac{1}{\rho } \frac{\partial P}{\partial y} \end{aligned}$$

2.6c $$\begin{aligned}&\frac{\partial w}{\partial t}+ u \frac{\partial w}{\partial x} +v\frac{\partial w}{\partial y} +w\frac{\partial w}{\partial z}-2 \Omega u =-\frac{1}{\rho } \frac{\partial P}{\partial z}-g. \end{aligned}$$

2.6d $$\begin{aligned}&\frac{\partial \rho }{\partial t}+u\frac{\partial \rho }{\partial x}+v\frac{\partial \rho }{\partial y}+w\frac{\partial \rho }{\partial z} +\rho \left( \frac{\partial u}{\partial x}+\frac{\partial v}{\partial y}+\frac{\partial w}{\partial z}\right) =0, \end{aligned}$$

2.6e $$\begin{aligned}&P=\rho {\mathcal {T}}, \end{aligned}$$

2.6f $$\begin{aligned}&\frac{\partial {\mathcal {T}}}{\partial t}+ u \frac{\partial {\mathcal {T}}}{\partial x} +v\frac{\partial {\mathcal {T}}}{\partial y} +w\frac{\partial {\mathcal {T}}}{\partial z} -\frac{\mu }{\rho }\left( \frac{\partial P}{\partial t}+ u \frac{\partial P}{\partial x} +v\frac{\partial P}{\partial y} +w\frac{\partial P}{\partial z} \right) =Q. \end{aligned}$$

where

$$\begin{aligned} \Omega =\frac{L'\Omega '}{U'}, \quad g=\frac{L'g'}{U'^2}=O(1), \quad \mu =\frac{{\mathfrak {R}}'}{c_p'}\approx 0.287, \quad Q=\frac{{\mathfrak {R}}' L'}{c_p' U'^3}Q'. \end{aligned}$$

Equations (2.6d) and (2.6e) lead to

$$\begin{aligned} \frac{1}{{\mathcal {T}}}\frac{D{\mathcal {T}}}{Dt}-\frac{\mu }{P}\frac{DP}{Dt}=0, \end{aligned}$$

where $\frac{D}{Dt}=\partial _t+u\partial _x+v\partial _y+w\partial _z$ is the material (or convective) derivative. Consequently, the (non-dimensionalised) potential temperature

2.7 $$\begin{aligned} \theta ={\mathcal {T}} \cdot \left( \frac{P_0}{P}\right) \end{aligned}$$

is conserved for each fluid parcel. (The potential temperature for a fluid parcel is the temperature that the parcel would attain if adiabatically brought to a reference pressure $P_0$, where $P_{0}$ is usually chosen to be 1, 000mb). Note that the potential temperature $\theta$ can be considered as an alternative variable for entropy (see [11]), hence relation (2.7) ensures that system (2.6) satisfies the second law of thermodynamics (see [1]).

The approach we employ for the construction of exact solutions to the system of governing equations (2.6) follows the viewpoint that, knowing the velocity field (*u*, *v*, *w*), the pressure *P*, and the density $\rho$ of a fluid parcel, the ideal gas law (2.6e) then prescribes the temperature ${\mathcal {T}}$ and the first law of thermodynamics (2.6f) identifies the heat sources. Dealing with heat sources is a particularly intricate modelling issue when air moisture and water vapour are involved [7–9]. However, matters are simplified when dealing with, for instance, mountain waves. These are gravity atmospheric waves resulting leeward from the crest of a mountain range whereby the air, which experiences a relatively uniform orographic lift up the mountain slope, develops a complicated downstream flow pattern corresponding to oscillations in the flow velocity, air temperature, and atmospheric pressure, see [12, 21, 25, 27, 28, 34]. The orographic lifting of air particles can (outside regions of active precipitation—see the discussions in [6, 18]) be reasonably considered a dry adiabatic process and so we set $Q\equiv 0$ in (2.6f) for the atmospheric wave motions being considered in this paper. As mentioned above, the compatibility condition (2.6f) then ensures that the prescribed atmospheric flow is consistent with both the first and second law of thermodynamics.

## Exact Solution

The general form of the exact and explicit solution of the governing equations (2.6) being considered is of the form

3.1 $$\begin{aligned} \begin{aligned}&x=\mathcal Ut+a-\frac{e^{-kb}}{k}\sin [k(a-ct)]\\&y=d+D(a,b)t \\&z=Z_0+\mathcal Wt- b+ \frac{e^{-kb}}{k}\cos [k(a-ct)]. \end{aligned} \end{aligned}$$

The parameters *c* and $k=2\pi /\lambda > 0$ denote the non-dimensionalised wave speed, and wavenumber, respectively (where $\lambda =\lambda '/L'$ is the non-dimensional wavelength). The velocity field determined by the solution (3.1) is obtained simply by taking the time derivative of the position variables in the Lagrangian formulation. Introducing $\xi =k(a-ct)$, and letting $\dot{(\cdot )}$ denote differentiation with respect to time, gives

3.2 $$\begin{aligned} \begin{aligned}&u=\dot{x}={\mathcal {U}}+{ce^{-kb}}\cos \xi \\&v=\dot{y}=D(a,b) \\&w=\dot{z}={\mathcal {W}}{+}c{e^{-kb}}\sin \xi . \end{aligned} \end{aligned}$$

The constants ${\mathcal {U}}, {\mathcal {W}}$ correspond to mean horizontal and vertical atmospheric currents (atmospheric gravity waves may propagate both vertically and horizontally on account of air compressibility and stratification). The function *D*(*a*, *b*) represents an atmospheric current which flows in the horizontal direction transverse to wave propagation, and which is prescribed in terms of the Lagrangian labelling variables (*a*, *b*) (see [2]) variables. Incorporating this transverse atmospheric current term in (3.1) further increases the potential of this exact solution to model physically interesting atmospheric flows, bearing in mind that, over sufficiently long length scales, all atmospheric gravity waves exhibit variations in a transverse direction. Indeed, even for relatively short mountain waves which arise when stable air flow passes over a mountain peak in a direction which is primarily perpendicular to the orientation of the mountain range, interesting variations of these waves in the transverse direction can often be observed. For instance, “atmospheric ship waves” (see the discussion in [34]) are waves formed in the lee of a mountain which exhibit transverse motion variations akin to the wake of a ship on the surface of water. Nice images of atmospheric ship waves are given in [21] for waves in the lee of the Beerenberg volcano on Jan Mayen island in the north Atlantic, and in [34] for waves along the Aleutian Island chain. Note also that mean background winds are typical in locations where mountain waves predominate, such as the Andes, the Alps, and the mountain ranges of Scandinavia, New Zealand and Antarctica, see [6, 14].

The form of the exact solution in (3.1) permits Lagrangian labelling variables $(a,d)\in {\mathbb {R}}^2$ without restriction from a mathematical perspective however, as discussed in Sect. 4, physical considerations will limit these variables depending on the atmospheric model being considered. The variable *b* is taken to be strictly positive to ensure that particle paths are non-self-intersecting in the (*x*, *z*)-plane and, for atmospheric wave solutions, limited in range: $0<b_0<b<b_1$. This range demarcates the vertical extent of the laminar flow region, outside of which turbulence is typically encountered, both beneath and aloft. Since $z_b<0$, the vertical coordinate *z* in (3.1) decreases with increasing *b*, and so $z(b_1,\cdot )<z(b,\cdot )<z(b_0,\cdot )$. Hence the exact solution (3.1) describes waves propagating through a laminar layer whose lower boundary corresponds to $b=b_1$, and upper boundary $b=b_0$. It can be inferred directly from (3.1) that oscillations of air parcels increase with ascending altitude, therefore wave motion is least noticeable along the lower boundary, and is most pronounced along the upper boundary. The constant $Z_0$ in (3.1) can be freely chosen as a fixed reference altitude, depending on the atmospheric flows being modelled.

**Fig. 1:**
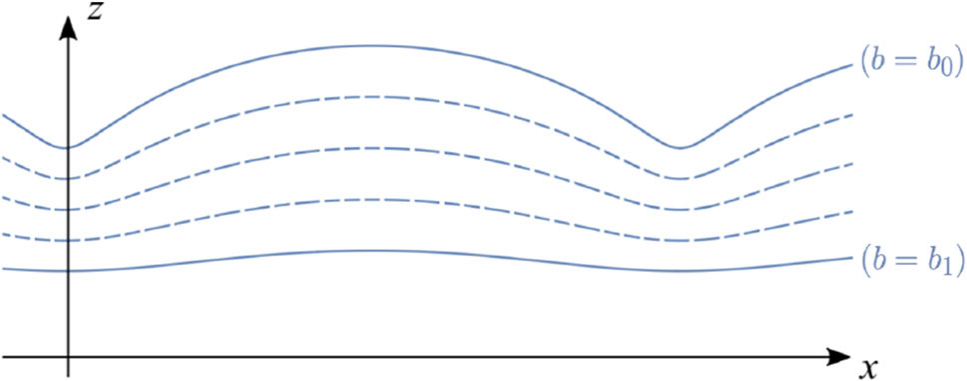
Particle paths for solution (3.1) viewed in the (*x*, *z*)-plane, and ignoring the horizontal and vertical background currents: ${\mathcal {U}},{\mathcal {W}}=0$. Air parcel trajectories correspond to trochoids, which are paths traced by points fixed in the interior of a disc, as the disc rolls without slipping along a horizontal line [3, 13]. The upper wavy boundary corresponds to $b=b_0$, while the lower less-wavy boundary corresponds to $b=b_1$, for $0<b_0<b_1$

For positive values of *b*, the oscillatory terms involving (*a*, *b*) in (3.1) prescribe trochoidal particle paths in the (*x*, *z*)-plane which have relatively wide crests, and narrow troughs, as illustrated in Fig. 1. This variance between the crest and trough regions is characteristic of strongly nonlinear waves, and contrasts with the regular sinusoidal features intrinsic to linear wave theory. We note that, in the limiting case as $b\downarrow 0$, the corresponding particle path is a cycloid which has a cusp at the wave trough, and it will be shown in Sect. 4 that the vorticity of the atmospheric flows being considered becomes unbounded in this limiting case. For negative values of *b*, the wave-like terms parameterised in terms of (*a*, *b*) Lagrangian variables in (3.1) prescribe self-intersecting prolate cycloids [13] in the (*x*, *z*)-plane, which cannot correspond to particle paths in atmospheric flows unless the flow is always three-dimensional (requiring $D(a,b)\ne 0$).

The Jacobian matrix of the coordinate transformation (3.1) is given by

3.3 $$\begin{aligned} \frac{\partial (x,y,z)}{\partial (a,d,b)}=\begin{pmatrix} 1-e^{-kb}\cos \xi &{} 0 &{} e^{-kb}\sin \xi \\ D_a(a,b)t &{} 1 &{} D_b(a,b)t \\ {-}e^{-kb}\sin \xi &{} 0 &{} {-}1{-}e^{-kb}\cos \xi \end{pmatrix}, \end{aligned}$$

whose determinant is

3.4 $$\begin{aligned} {\mathfrak {D}}=e^{-2kb}{-}1<0. \end{aligned}$$

Since the determinant ${\mathfrak {D}}$ in (3.4) is independent of time, the mapping defined by (3.1) must be volume preserving and hence

3.5 $$\begin{aligned} u_x+v_y+w_z=0. \end{aligned}$$

This relation, coupled with the assumption that the density $\rho$ is of the form $\rho (a,d,b)=\rho (b)$, implies that (2.6d) holds. To see this, first consider a density dependence on the (*x*, *y*, *z*, *t*) variables of the form $\rho =f(X,Y,Z)$ where $X=x-({\mathcal {U}}+c)t=(\xi -e^{-kb}\sin \xi )/k$, $Y=y-D(a,d)t=d$, and $Z=z-Z_0-Wt=\left( e^{-kb}\cos \xi \right) /k$. Then $\rho =f(X,Y,Z)$ corresponds to a dependence on Lagrangian labelling variables of the form $\rho =\rho (\xi ,d,b)$, with

$$\begin{aligned} \rho _d=f_X X_d+f_YY_d+f_ZZ_d=f_Y. \end{aligned}$$

Hence $f_Y\equiv 0$ if $\rho$ is independent of *d*. In this case $\rho =\rho (\xi ,b)=f(X,Z)$, which leads to

3.6 $$\begin{aligned} \rho _{\xi }&=f_X X_{\xi }+f_ZZ_{\xi }=\frac{1-e^{-kb}\cos \xi }{k}f_X-\frac{e^{-kb}\sin \xi }{k}f_Z \nonumber \\&=-\frac{1}{ck}\left( \left( u-\left( {\mathcal {U}}+c\right) \right) f_X+\left( w-W\right) f_Z\right) =-\frac{1}{ck}\frac{df}{dt}=-\frac{1}{ck}\frac{D\rho }{Dt}. \end{aligned}$$

Therefore, assuming that the density is of the form $\rho =\rho (b)$, equations (3.5) and (3.6) imply that (2.6d) holds. Bearing in mind $z_b<0$, we assume that the density is an increasing function of the labelling variable *b*, of the form $\rho =\rho (b)$ with $\rho '(b)\ge 0$. The inverse of the Jacobian matrix (3.3) can be calculated to get

3.7 $$\begin{aligned} \frac{\partial (a,d,b)}{\partial (x,y,z)}= \frac{1}{1-e^{-2kb}} \begin{pmatrix} 1{+}e^{-kb}\cos \xi &{} 0 &{} e^{-kb}\sin \xi \\ t {\mathcal {D}}_1 &{} 1-e^{-2kb} &{} t {\mathcal {D}}_2 \\ -e^{-kb}\sin \xi &{} 0 &{} e^{-kb}\cos \xi -1 \end{pmatrix}, \end{aligned}$$

where we denote

$$\begin{aligned} {\mathcal {D}}_1&=\left[ D_a ({-}1{-}e^{-kb}\cos \xi )+ D_b e^{-kb}\sin \xi \right] {\mathcal {D}}_2&=\left[ D_b (1-e^{-kb}\cos \xi ) -D_a e^{-kb}\sin \xi \right] . \end{aligned}$$

It follows that the density decreases as a function of height, since

3.8 $$\begin{aligned} \frac{\partial \rho }{\partial z} = \frac{d \rho }{d b} \frac{\partial b}{\partial z}=-\rho '(b)\frac{1-e^{-kb}\cos \xi }{1-e^{-2kb}}\le 0, \end{aligned}$$

for *b* positive. Furthermore, we can explicitly compute

$$\begin{aligned} \begin{pmatrix} u_x &{} u_y &{} u_z \\ v_x &{} v_y &{} v_z \\ w_x &{} w_y &{} w_z \end{pmatrix} = \begin{pmatrix} u_a &{} u_d &{} u_b \\ v_a &{} v_d &{} v_b \\ w_a &{} w_d &{} w_b \end{pmatrix} \begin{pmatrix} a_x &{} a_y &{} a_z \\ d_x &{} d_y &{} d_z \\ b_x &{} b_y &{} b_z \end{pmatrix} = \frac{1}{{\mathfrak {D}}} \begin{pmatrix} -e^{-kb}\sin \xi &{} 0 &{} kce^{-kb} \left[ \cos \xi -e^{-kb}\right] \\ - {\mathcal {D}}_1 &{} 0 &{} -{\mathcal {D}}_2 \\ kce^{-kb} \left[ \cos \xi +e^{-kb}\right] &{} 0 &{} kce^{-kb} \sin \xi \end{pmatrix}. \end{aligned}$$

The vorticity vector $\varvec{\omega }=\nabla \times \varvec{u}=(w_y-v_z,u_z-w_x,v_x-u_y)$ for the flow prescribed by (3.1) is given by

3.9 $$\begin{aligned} \varvec{\omega }=\frac{1}{{\mathfrak {D}}} \left( {\mathcal {D}}_2,-2kce^{-2kb}, - {\mathcal {D}}_1\right) . \end{aligned}$$

It follows from (3.4) and (3.9) that the vorticity becomes unbounded as $b\downarrow 0$. The flow vorticity $\varvec{\omega }$ is three-dimensional if *D*(*a*, *b*) is non-constant. For *D* constant, the vorticity reduces to the one-dimensional expression $\omega =-2kce^{-2kb}/{\mathfrak {D}}$.

The material acceleration of air parcels can be explicitly computed by taking the time derivative of the Lagrangian formulation of the velocity field (3.2), leading to

3.10 $$\begin{aligned} \frac{Du}{Dt}=kc^2e^{-kb}\sin \xi , \ \frac{Dv}{Dt}=0, \ \frac{Dw}{Dt}={-}kc^2{e^{-kb}}\cos \xi . \end{aligned}$$

Thence the Euler equations (2.6a)–(2.6c) can be expressed as

3.11 $$\begin{aligned} P_{x}&=-\rho (b)e^{-kb}\sin \xi \left( kc^2+ 2\Omega c \right) -\rho (b)2\Omega {\mathcal {W}}, \quad P_y=0, \nonumber \\ P_{z}&=\rho (b) {e^{-kb}}\cos \xi \left( kc^2+2\Omega c \right) +\rho (b)2\Omega {\mathcal {U}} -g\rho (b). \end{aligned}$$

Computing $$ \begin{pmatrix} P_a&P_d&P_b \end{pmatrix}=\begin{pmatrix} P_x&P_y&P_z \end{pmatrix}\cdot \frac{\partial (x,y,z)}{\partial (a,d,b)}$$ from (3.3) and (3.11) gives

3.12 $$\begin{aligned} P_a&=-\rho (b)e^{-kb}\sin \xi \left( kc^2+ 2\Omega c +2\Omega {\mathcal {U}}-g \right) +2\Omega {\mathcal {W}}\rho (b) \left[ e^{-kb}\cos \xi -1\right] , \end{aligned}$$

3.13 $$\begin{aligned} P_d&=0, \end{aligned}$$

3.14 $$\begin{aligned} P_b&= -\rho (b)e^{-2kb}\left( kc^2+ 2\Omega c \right) -\rho (b) {e^{-kb}}\cos \xi \left( kc^2+2\Omega c +2\Omega {\mathcal {U}} -g \right) \nonumber \\&\qquad -\rho (b)2\Omega {\mathcal {W}} e^{-kb}\sin \xi -\rho (b)2\Omega {\mathcal {U}} +g\rho (b) \end{aligned}$$

In order for (3.1) to be a valid solution of (2.6), it is necessary that mixed derivatives of the pressure match. From (3.12) and (3.14), respectively, we compute

3.15 $$\begin{aligned} P_{ab}&=\left[ k\rho (b)e^{-kb} -\rho '(b)e^{-kb}\right] \sin \xi \left( kc^2+ 2\Omega c +2\Omega {\mathcal {U}}-g \right) \nonumber \\&\quad -\rho '(b)2\Omega {\mathcal {W}} +2\Omega {\mathcal {W}}\left[ \rho '(b)-k\rho (b)\right] e^{-kb}\cos \xi . \end{aligned}$$

3.16 $$\begin{aligned} P_{ba}&= -\rho (b)2\Omega {\mathcal {W}} k e^{-kb}\cos \xi +k\rho (b) {e^{-kb}}\sin \xi \left( kc^2+2\Omega c +2\Omega {\mathcal {U}} -g \right) . \end{aligned}$$

Assuming non-constant density ($\rho '\not \equiv 0$), the expressions (3.15) and (3.16) can match only if either ${\mathcal {W}}=0$, or $\Omega =0$. The resulting solutions represent two fundamentally different atmospheric gravity wave flows, which we now address separately.

## Nonlinear Atmospheric Gravity Wave Solutions

### Atmospheric Billows in the Equatorial *f*-Plane ($\Omega \ne 0$, ${\mathcal {W}}=0$)

If we retain the Coriolis effects of Earth’s rotation in the nonlinear governing equations (2.6) (taking $\Omega \ne 0$) then equality holds between the mixed partial derivatives (3.15) and (3.16) only if

4.1 $$\begin{aligned} kc^2+2\Omega c +2\Omega {\mathcal {U}} -g=0, \quad \text{ and } \quad {\mathcal {W}}=0. \end{aligned}$$

The condition ${\mathcal {W}}=0$ implies that the atmospheric flow has no mean vertical velocity component, and hence it is not possible to model upward propagating mountain waves in the Equatorial *f*-plane approximation. Instead, the exact solution (3.1) which satisfies the relations in (4.1) prescribes an atmospheric flow which is characteristic of trapped lee waves, whereby air parcels propagate horizontally while oscillating vertically about a fixed mean altitude.

Trapped lee waves are mountain waves which are trapped in a stable layer in the lower troposphere at altitudes close to the mountain peak. These gravity waves entrain atmospheric motion which corresponds qualitatively to the particle paths illustrated in Fig. 1, whereby wave motion is almost imperceptible in the lower part of the laminar layer which lies beneath the mountain peak. Closer to ground level in the planetary boundary layer the regular wave pattern is distorted by frictional effects, and we do not seek to model this region of atmospheric flow. Trapped lee winds can generate lenticular clouds on the leeward side of the mountain, while a cap cloud may be generated directly over the mountain peak [6, 21].

Denoting ${\tilde{g}}= g-2\Omega {\mathcal {U}}$, then (4.1) implies that $P_a\equiv 0$ and equation (3.14) can be integrated to give the pressure distribution

$$\begin{aligned} P(b)={\tilde{P}}_0+{\tilde{g}}\int _{b_1}^{b} \rho ({\tilde{b}})\left( 1-e^{-2k{\tilde{b}}}\right) d{\tilde{b}}, \end{aligned}$$

where ${\tilde{P}}_0=P(b_1)$ is a reference value for the pressure at the lower boundary of the laminar layer given by $b=b_1$. It follows from the third equation in (3.11) that

$$\begin{aligned} P_{z} =-{\tilde{g}} \rho (b)\left( 1- {e^{-kb}}\cos \xi \right) \le 0, \end{aligned}$$

which, combined with (3.8), establishes that both the pressure and density decrease with altitude. The decrease of air density with height increases the amplitude of oscillations aloft, as illustrated in Fig. 1.

Regarding the atmospheric temperature distribution ${\mathcal {T}}$ corresponding to the atmospheric motion prescribed by the exact solution (3.1) which satisfies the dispersion relation in (4.1), note that the equation of state (2.6e) implies that ${\mathcal {T}}={\mathcal {T}}(b)$, since $\rho =\rho (b)$ and $P=P(b)$. Therefore the temperature of an air parcel does not change during its motion, and so the first law of thermodynamics (2.6f) must hold.

The first equality in (4.1) corresponds to a dispersion relation expressed in terms of non-dimensionalised physical parameters, giving

4.2 $$\begin{aligned} c=\frac{\pm \sqrt{k{\tilde{g}}+\Omega ^2}-\Omega }{k}. \end{aligned}$$

These solutions correspond to two possible non-dimensional wave speeds, one corresponding to right-moving, and the other to left-moving, atmospheric waves (with the latter having a slightly larger magnitude due to the presence of the non-dimensionalised rotational speed $\Omega =7.3\times 10^{-3}$ rad). In the Equatorial region where the trade winds (or easterlies) prevail, we may take the negative value $c<0$ and restrict the Lagrangian labelling *a*-variable to the range $a\in (-\infty , 0]$, with the wave taken to originate at the mountain peak located at $a=0$. Note that the Lagrangian labelling variable *d* will also be restricted in range, $d\in [-d_0,d_0]$ for some $d_0$, since the *f*-plane approximation in the Euler equations (2.6a)–(2.6c) is applicable to Equatorial regions with limited latitudinal variation.

For atmospheric gravity waves whose wavelengths are of the order of 2 km (a length-scale characteristic of mountain waves which was assumed in the non-dimensionalisation procedure leading to the governing equations (2.6)) the ${\tilde{g}}$ term will be several orders of magnitude larger than those involving $\Omega$, in which case $c\approx \pm \sqrt{g/k}$. Reversing the non-dimensionalisation process invoked in Sect. 2 results in a dispersion relation for dimensional variables of the exact same form as (4.1) and (4.2) above, if we replace all parameters by their dimensional (‘primed’) versions, and with *c* and ${\mathcal {U}}$ given by $c'=U'c$ and ${\mathcal {U}}' =U' {\mathcal {U}}$, respectively.

### Upward Propagating Mountain Waves in Non-rotating Coordinates ($\Omega =0$, ${\mathcal {W}}\ne 0$)

In order to model vertically propagating atmospheric waves (for which ${\mathcal {W}}\ne 0$) the effects of the Earth’s rotation must be neglected by setting $\Omega =0$ in the governing equations (2.6), and in the subsequent considerations of Sects. 2 and 3. The resulting simplification in the governing equations (2.6) leads to a greater flexibility for the exact solution (3.1) to model nonlinear atmospheric gravity waves, since all prior considerations are now applicable at arbitrary latitudes (being no longer restricted to the Equatorial region), and the coordinate system may be oriented with full horizontal generality (with the *x*- and *y*-axes being no longer restricted to pointing in the zonal and meridional directions, respectively). Note that, in this setting, solution (3.1) generalises an exact solution for upward propagating mountain waves recently derived in [6] through incorporating transverse currents by way of the *D*(*a*, *b*) term.

For $\Omega =0$, mixed pressure derivatives (3.15) and (3.16) reduce to

4.3 $$\begin{aligned}&P_{ab} =\left( kc^2 -g \right) \left[ k\rho (b)e^{-kb} -\rho '(b)e^{-kb}\right] \sin \xi , \end{aligned}$$

4.4 $$\begin{aligned}&P_{ba}= k\rho (b)\left( kc^2 -g \right) {e^{-kb}}\sin \xi . \end{aligned}$$

Assuming non-constant density ($\rho '\not \equiv 0$), relation (4.3) and (4.4) match only when the non-dimensional dispersion relation $c^2=g/k$ holds, which can be solved to get the non-dimensional wavespeed

4.5 $$\begin{aligned} c=\pm \sqrt{g/k}. \end{aligned}$$

Parameterising the leeward direction by $a\in [0,\infty )$, with the mountain peak located at $a=0$, we choose the positive value $c>0$ in (4.5) above. Note that the dimensional form of the dispersion relation, and associated wavespeed, takes the same form as those above if we replace *g*, *k* by $g',k'$, respectively, and *c* by the dimensionalised wavespeed $c'=U'c$.

Relation (4.5) implies that $P_a\equiv 0$, and the corresponding pressure distribution then takes the form

$$\begin{aligned} P(b)=P_0+g\int _{b_1}^{b} \rho ({\tilde{b}})\left( 1-e^{-2k{\tilde{b}}}\right) d{\tilde{b}}, \end{aligned}$$

where $P_0=P(b_1)$ is a reference value for the pressure at $b=b_1$ at the lower boundary of the laminar layer. It follows from the third equation in (3.11) that

$$\begin{aligned} P_{z}=g\rho (b) \left( e^{-kb}\cos \xi -1\right) \le 0. \end{aligned}$$

This relation, together with (3.8), shows that both the pressure and density decrease with altitude, as required, since upward propagating mountain waves occur when the air temperature and density decrease above the mountain peak. In this setting—and when there is no thermal inversion layer, or unstable layer with high-speed winds, aloft—there is a noticeable escalation in wave amplitude with increasing altitude, with waves propagating upwards into the lower stratosphere (unless they steepen and break before reaching the tropopause). These qualitative features of motion are captured by the exact solution (3.1) (as illustrated in Fig. 1, except now tilted upwards to allow for ${\mathcal {W}}>0$ in these anabatic waves) whereby the associated oscillations of air particles increase with ascending altitude: wave motion is most pronounced along the upper boundary, and least noticeable along the lower boundary. Breaking waves aloft generate strong vertical currents (in excess of 30m s^-1^) and clear air turbulence and vortices (which are a significant hazard to aviation) may also develop. This singular behaviour is captured to some extent by the exact solution (3.1) in the sense that, as $b_0\rightarrow 0$, the wave amplitude at the upper layer is maximised and the vorticity (3.9) becomes unbounded, which is indicative of the presence of strong vortices aloft.

Regarding the atmospheric temperature distribution ${\mathcal {T}}$ corresponding to the atmospheric motion prescribed by the exact solution (3.1) which satisfies the dispersion relation (4.5), note that the equation of state (2.6e) implies that ${\mathcal {T}}={\mathcal {T}}(b)$, since $\rho =\rho (b)$ and $P=P(b)$. Therefore the temperature of an air parcel does not change during its motion, and so the first law of thermodynamics (2.6f) must hold.

#### On Downward Propagating Lee Waves

We remark that it would be natural to try to adapt the exact solution (3.1) to model waves propagating downwards on the lee side of a mountain and, as a first step in this direction, we note that one can freely choose ${\mathcal {W}}<0$ in the exact solution (3.1) in order to model a downdraft. However, a defining characteristic of katabatic waves is the existence of rotors (overturning eddies) indicating low-level turbulence beneath the laminar layer, and in order for (3.1) to model such a phenomenon one would expect the most pronounced wave motion to occur at the lower boundary of the laminar layer (rather than at the top) with the velocity field tending towards an unbounded vorticity (thus indicating wave-breaking, and strong vortices) at the lower boundary of the laminar layer. This reversal in the direction of wave amplification, with amplitudes increasing as one descends from the top layer to the bottom, can be achieved in (3.1) if one reverses the polarity of the first *b* term appearing in the description of the *z*-variable, defining the vertical variable instead by ${\hat{z}}=Z_0+\mathcal Wt+b+ (e^{-kb}\cos \xi )/k$. However, unfortunately this attempt to capture downward propagating mountain waves breaks down irretrievably at the dispersion relation stage, which now reads $c^2=-g/k$. This dispersion relation cannot define any real-valued speed for atmospheric gravity waves.
